# Stable microbial community composition on the Greenland Ice Sheet

**DOI:** 10.3389/fmicb.2015.00193

**Published:** 2015-03-20

**Authors:** Michaela Musilova, Martyn Tranter, Sarah A. Bennett, Jemma Wadham, Alexandre M. Anesio

**Affiliations:** ^1^School of Geographical Sciences, University of Bristol, BristolUK; ^2^NERC Isotope Geosciences Laboratory, British Geological SurveyNottingham, UK

**Keywords:** Greenland Ice Sheet, 16S rRNA, aeolian inputs, snow, stable carbon isotopes, cryoconite

## Abstract

The first molecular-based studies of microbes in snow and on glaciers have only recently been performed on the vast Greenland Ice Sheet (GrIS). Aeolian microbial seeding is hypothesized to impact on glacier surface community compositions. Localized melting of glacier debris (cryoconite) into the surface ice forms cryoconite holes, which are considered ‘hot spots’ for microbial activity on glaciers. To date, few studies have attempted to assess the origin and evolution of cryoconite and cryoconite hole communities throughout a melt season. In this study, a range of experimental approaches was used for the first time to study the inputs, temporal and structural transformations of GrIS microbial communities over the course of a whole ablation season. Small amounts of aeolian (wind and snow) microbes were potentially seeding the stable communities that were already present on the glacier (composed mainly of Proteobacteria, Cyanobacteria, and Actinobacteria). However, the dominant bacterial taxa in the aeolian samples (Firmicutes) did not establish themselves in local glacier surface communities. Cryoconite and cryoconite hole community composition remained stable throughout the ablation season following the fast community turnover, which accompanied the initial snow melt. The presence of stable communities in cryoconite and cryoconite holes on the GrIS will allow future studies to assess glacier surface microbial diversity at individual study sites from sampling intervals of short duration only. Aeolian inputs also had significantly different organic δ^13^C values (-28.0 to -27.0‰) from the glacier surface values (-25.7 to -23.6‰), indicating that *in situ* microbial processes are important in fixing new organic matter and transforming aeolian organic carbon. The continuous productivity of stable communities over one melt season makes them important contributors to biogeochemical nutrient cycling on glaciers.

## Introduction

Glaciers and ice sheet ecosystems are dominated by microbes and are sites of active biogeochemical cycling. Considering that they cover ∼11% of the global surface (Benn and Evans, 2010), these icy ecosystems have recently been demonstrated to have regional and global impacts worthy of being recognized as a biome (Anesio and Laybourn-Parry, 2012). High microbial activity is concentrated in glacier surface debris called cryoconite (Tranter et al., 2004; Hodson et al., 2005). Accumulation of the dark organic and inorganic cryoconite causes enhanced melting around the deposited sediment (Gerdel and Drouet, 1960; Takeuchi et al., 2001). This leads to the formation of water-filled depressions with cryoconite at the base (often between 1 cm and 1 m in diameter and usually 1 cm and 0.5 m deep), called cryoconite holes (Fountain et al., 2004). Microbes in cryoconite holes are important contributors to biogeochemical cycling on the surface of glaciers with a potential to fix as much as 64 Gg of carbon per year on a global basis (Anesio et al., 2009). They are therefore considered to be a significant source of bioavailable carbon and nutrients to downstream subglacial and coastal ecosystems (Anesio et al., 2009; Stibal et al., 2012). The microbial activity in cryoconite is also believed to cause a further darkening of the already dark inorganic particulates in cryoconite by producing and/or transforming organic carbon (Takeuchi et al., 2001; Takeuchi, 2002; Hodson et al., 2010a). This can lead to a significant decrease in glacier surface albedo and thereby increased melt rates, considering cryoconite covers 0.1–10% of the ablation zone of glaciers (Hodson et al., 2007, 2010a).

Supraglacial (glacier surface) cryoconite and cryoconite hole water host abundant and diverse viral, prokaryotic, and eukaryotic life forms (Sawstrom et al., 2002; Anesio et al., 2007; Edwards et al., 2011). To date, there have been very few culture independent studies of microbial diversity in cryoconite. Molecular analyses have focused on the cryoconite of glaciers in Svalbard, the Alps, and Antarctica (Christner et al., 2003; Foreman et al., 2007; Edwards et al., 2013a,b; Zarsky et al., 2013). Greenland Ice Sheet (GrIS) cryoconite samples were studied for the first time using T-RFLP analysis of rRNA genes by Cameron et al. (2012b), when they compared them against samples from different locations in the Arctic and Antarctica. They found considerable diversity between cryoconite hole communities, whose structure varied with geography (Cameron et al., 2012b). Similarly, Edwards et al. (2014) compared GrIS cryoconite hole bacterial community structures to those from alpine glaciers and Svalbard using Terminal restriction fragment length polymorphism. The authors found that local and regional environmental conditions impact significantly on the community structures, compositions, and metabolomes (Edwards et al., 2014). Recently, Stibal et al. (2015) used 454 pyrosequencing of the 16S rRNA gene and 16S rRNA cDNA to compare cryoconite hole diversity at the margin and in the interior of GrIS. They observed significant differences between bulk (rDNA) and potentially active (rRNA) communities from the two sites (Stibal et al., 2015).

Cryoconite microbial diversity differs considerably from nearby ice-marginal habitats in Svalbard (Edwards et al., 2013c). Glacier specific factors (Edwards et al., 2011, 2013a,c) are postulated to influence the cryoconite community diversity. The significant variation in cryoconite microbial taxa between glaciers suggests that the communities develop individually upon localized aeolian and/or glacial melt transportation to glaciers (Cameron et al., 2012b). Airborne microbes from local and distant sources are also thought to be deposited in snowpacks on glaciers through ice crystal nucleation and subsequent snowfall (Xiang et al., 2009; Larose et al., 2010; Harding et al., 2011). Their community composition varies with altitude (Yoshimura et al., 1997) and the distribution of local biological material (Liu et al., 2009). Cameron et al. (2014) performed the first molecular-based studies describing the microbial diversity in snow on western portions of the vast GrIS. Their results indicate that snow from western regions of GrIS contains microbial assemblages, which were aerosolized from distant soil sources, transported in the atmosphere, and co-precipitated with the snow (Cameron et al., 2014). Furthermore, Stibal et al. (2015) hypothesized that both local sources and long distance transport contribute to the gene pool of marginal sites on the GrIS, which had higher bulk alpha diversity than the interior of GrIS. It remains to be determined whether this is also the case for other locations on the GrIS and whether supraglacial microbial communities are indeed structured by aeolian inputs from proximal habitats (Edwards et al., 2014).

Stable carbon isotope (δ^13^C) variations in prokaryotic and eukaryotic life forms result from isotope variations in source CO_2_ and microbes adopting various metabolic/carbon fixation pathways that fractionate carbon to varying extents (Fogel and Cifuentes, 1993). The carbon isotope composition of CO_2_(g) is relatively constant across the globe with an approximate average value of -8‰; (Cuntz, 2011), whereas the carbon isotope values of dissolved inorganic carbon used by cryoconite microorganisms may be higher (∼0‰; Fogel and Cifuentes, 1993). The degree of isotope fractionation from the source value depends on which carbon fixation pathway is being used. For example the predominant carbon fixation pathway for photoautotrophs is the Calvin-Benson-Bassham (CBB) cycle with Rubisco enzyme resulting in a -20 to -30‰ fractionation (Park and Epstein, 1960; Berg et al., 2010). Other pathways result in fractionations as small as -0.2‰; (Berg et al., 2010). In addition to autotrophs, heterotrophs will feed off the supply of organic matter resulting in a +1‰ increase in δ^13^C values (Deniro and Epstein, 1978). Therefore the location and varying composition of microbial communities may result in distinct δ^13^C values that could be used to identify organic carbon inputs and/or community changes over time.

Hitherto, hardly any studies have assessed the evolution of the structure of these microbial communities during a summer melt season, despite the fact that large differences have been found in their diversity between two different sampling periods over the same season in Svalbard (Larose et al., 2010). This is clearly important for studies of microbial diversity on glaciers, since if there is marked temporal variations in community structure, sampling needs to be conducted over long timescales to capture the true microbial diversity accurately. Hence, in this study, a range of experimental approaches was used for the first time to study the inputs and transformations of GrIS supraglacial microbial communities over the course of a whole ablation season. These include dust traps and snow sampling set up to characterize the contribution of microbes of aeolian origin. The periodic collection of glacier surface cryoconite hole, dispersed supraglacial cryoconite, and dust trap samples allowed for the seasonal component of GrIS surface microbial community dynamics to be determined. Stable isotope analyses (δ^13^C) were also used for the first time to investigate the origin and transformation of organic carbon at the surface of glaciers.

## Materials and Methods

### Field Sampling Strategy

#### Snow and Glacier Surface Samples

Sampling was conducted on Leverett Glacier (∼67.10^∘^N, 50.20^∘^W), which is the southern lobe of the Russell Glacier in the southwestern region of GrIS. The sampling site was a delimited circular area 8 m in diameter, chosen randomly ∼2 km from the southwest margin of Leverett Glacier. Dispersed cryoconite debris on the glacier surface [‘dirty ice (DI)’] and cryoconite hole debris were sampled once every 10–14 days (a sampling time point) during the 2012 ablation season (15th May, 28th May, 11th June, 25th June, 9th July, 23rd July, 1st August). Cryoconite hole debris was collected aseptically with furnaced spatulas (550^∘^C for 4 h) into sterile 1L Whirl-Pak bags (Nasco). Non-lidded cryoconite holes were sampled that were 10–20 cm in size, 5–10 cm deep, and with particle sizes <3 mm. This was done to ensure the cryoconite hole microbial communities sampled would be similar enough to each other to be pooled together into one sample per time point. All cryoconite holes with those characteristics were sampled at each time point within the delimited sampling site, collecting ∼3–10 g wet weight per cryoconite hole proportionally to their size. Efforts were made to collect the same amount of sample from each cryoconite hole at every time point. Greater amounts of debris were not collected to avoid disrupting microbial communities within the holes. The debris was pooled together for each sampling time point to secure sufficient sample material for the subsequent analyses (∼50 g wet weight per time point). Edwards et al. (2011) previously demonstrated an absence of spatial patterns in Arctic cryoconite hole microbial communities at intraglacier scales (with 1 km spaced sampling over a 2 year period). It was therefore presumed that the impact of pooling very similar and neighboring cryoconite hole debris would be minimal within the small sampling site, as has been performed previously (Edwards et al., 2014; Stibal et al., 2015). Samples were transferred to the field camp laboratory for frozen storage. They were transported frozen in insulated containers to the University of Bristol for storage at -25^∘^C prior to laboratory analyses. Samples for microbial abundance analyses (see Microbial Abundance) were fixed with 2% v/v 0.22 μm pre- filtered glutaraldehyde in sterile 50 mL centrifuge tubes.

Snow samples were collected on May 13th, before snowmelt had occurred in the sampling site. The temperatures began to rise in the sampling area on the next day and all of the surface snow turned to slush by the 15th May, before melting away by 20th May. DI and snow samples were collected aseptically into 5 L Whirl-Pak bags (Nasco). Great care was taken to first systematically remove the top 5 cm of the snow with a sterile spatula (as above), before collecting the snow beneath to avoid contamination (Amato et al., 2007; Larose et al., 2010). The DI had particle sizes <1 mm and was present in patches within the sampling area. As for the cryoconite holes, the DI samples were chosen so that they would be similar enough to each other to be pooled together into one sample per time point. All DI samples were collected at each time point within the delimited sampling site, collecting ∼20 g of ice/debris mixture per patch. Greater amounts of debris were not collected to avoid disrupting microbial communities in the debris, as above. The debris was pooled together for each sampling time point to secure sufficient sample material for the subsequent analyses once melted and filtered (∼30 g wet weight per time point). The snow and DI samples were melted at ambient temperature (∼10^∘^C) upon transportation to the field laboratory tent. They were filtered immediately through a sterile, pre-cleaned glass filtration apparatus with sterile filters. The glass apparatus was sterilized by first rinsing 6x with Milli-Q water (18.2 MΩ cm^-1^ deionized water, filtered through 0.22 μm membranes) and then furnacing at 550^∘^C for 4 h. Six filters per sample were prepared: 3x pre-furnaced GF/F (Millipore) for stable carbon isotope analysis and 3x 0.22 μm 47-mm filters (Millipore) for microbial abundance analyses, both described below. Sample filters were stored in sterile, pre-furnaced (550^∘^C for 4 h) aluminum foil. Procedural blanks were carried out by filtering autoclaved Milli-Q water using the same procedure as for the samples. All filters were immediately frozen in the field laboratory freezer. As above, they were transported frozen in insulated containers to the University of Bristol for storage at -25^∘^C prior to laboratory analyses. Filters used for microbial abundance analyses were fixed with glutaraldehyde, as above, prior to transportation to the University of Bristol.

#### Dust Traps

Six dust traps, based on the design by (Lancaster, 2002), were set up in a hexagonal shape (3 m in diameter) in the center of the sampling site. They consisted of 2 m long metal poles (5 cm wide) within 1 m long plastic pipes (10 cm wide), mounted with Bundt cake pans (25 cm wide and 10 cm deep). Each pan had a 7 cm opening in the center, through which a metal pole could fit. The poles were placed into 1.5 m deep holes drilled into the glacier surface, which ablated at a rate of ∼1 m/week. New holes needed to be re-drilled for the poles after each sampling time point. Pans remained at a constant height of 1 m above the surface by resting on top of the plastic pipes on the outside of the metal poles. Each pan had a metal mesh (stainless steel low-carbon woven wire with a 3.33 mm hole diameter) emplaced 4 cm below the rim, containing a 1 cm layer of borosilicate glass beads (Sigma–Aldrich). The beads within the mesh created a rough surface to aid the capture of aeolian dust in the pans (around the beads and below the mesh) and to prevent the dust from being blown away from the dust traps. To sterilize the pans (stainless steel with an anti-adhesive Teflon coating), they were first rinsed 6x with Milli-Q water, acid-washed for 24 h, before being wrapped in pre-furnaced aluminum foil, autoclaved, and stored in sterile 5 L Whirl-Pak bags prior to installation within the dust traps. The mesh and the beads were washed 6x with Milli-Q water, acid-washed for 24 h, furnaced (550^∘^C for 4 h), wrapped in pre-furnaced aluminum foil, and stored in sterile 5 L Whirl-Pak bags until they were mounted into the dust traps.

The dust traps were put in place during the first sampling time point 0 (15th May, 2012). Thereafter, they were removed aseptically, wrapped in pre-furnaced aluminum foil, stored in sterile 5 L Whirl-Pak bags, and transported to the field laboratory. All components of the dust trap (pan, mesh, and marbles) were washed 6x with autoclaved and 0.22 μm filtered Milli-Q water, which was collected in sterile 5 L Whirl-Pak bags. The water-sample mixture was filtered using the sterile filtration procedure (including procedural blanks) and stored as the filtered samples in section “Snow and Glacier Surface Samples.” Similarly, the filters were fixed with glutaraldehyde for microbial abundance analyses as above (see Snow and Glacier Surface Samples). Subsequently, the dust trap components were wrapped in pre-furnaced aluminum foil during transit, then unwrapped, and remounted back onto the poles until the next sampling time point, when the procedure was repeated.

### Microbial Diversity Analyses

#### DNA Extraction

DNA was extracted from the cryoconite hole, DI, snow, and dust trap samples using a variation of the Pace & Spear laboratory phenol–chloroform protocol (Dojka et al., 1998). This method was used instead of the more commonly used Powersoil DNA extraction kit (MoBio) for cryoconite studies because of the very low DNA content available from the dust traps. Negative controls were used throughout the entire DNA extraction process. In short, 1.0 g of cryoconite hole debris or one sample filter was added aseptically to a sterile 2 mL bead-beating tube containing 0.3 g of lysis beads (0.7 mm sized garnets). The samples were mixed by inversion with 500 μl of 0.22 μm filter sterilized buffer A [200 mM Tris (pH 8.0–8.5), 20 mM EDTA, 200 mM NaCl] and 200 μl of 0.22 μm filter sterilized 20% sodium dodecyl sulfate (SDS). They were incubated at 37^∘^C for 30 min, followed by the addition of 500 μl phenol–chloroform–isoamyl alcohol (at a ratio of 25:24:1 buffered to ∼pH 8) and horizontal vortexing for 10 min. Supernatant was transferred to a new tube after centrifugation at maximum speed for 4 min. The same extraction steps were repeated again with phenol–chloroform–isoamyl, except that the mixture was mixed thoroughly by inversion instead of by vortexing for 10 min. After the supernatant was removed the second time, DNA was precipitated by the addition of 0.22 μm filter sterilized 60 μl of 3 M sodium acetate and 600 μl ice-cold 100% isopropanol. The samples were incubated at -20^∘^C for 24 h, followed by centrifugation at maximum speed for 30 min. The pellet was washed twice with 500 μl of 100% ethanol and resuspended in 30 μl of 0.22 μm filter-sterilized 10 mM Tris buffer (pH 8.0). Samples were stored at -80^∘^C prior to further analyses.

#### 16S rRNA Gene Barcoded Sequencing

In total, 14 DNA samples and two negative controls were selected and subjected to Ion Torrent amplicon sequencing. Six cryoconite hole (C) and six DI samples were chosen from time points spanning the whole ablation season (labeled as 0,1,2,3,4, and 6). There was a very limited amount of DNA in the dust trap samples. Therefore, samples from all time points had to be pooled into one sample for 16S rRNA analysis (DT). Therefore, we cannot ascertain whether or not there is seasonality in the microbial and organic carbon input from the dust. Snow (SN) was only present at the start of the season, which was also pooled into one sample to provide enough DNA for sequencing. Standard barcoded bacterial V3 16S rRNA gene primers were used: 341F primer (CCTACGGGAGGCAGCAG) and 518R (ATTACCGCGGCTGCTGG; Muyzer et al., 1993). They had four base tags added to the 5^′^end of the primers, which were used in a combination of 4 forward × 4 reverse primers to create barcodes unique to each sample. The 16S rRNA gene was amplified using a Finnzymes Phusion Taq polymerase and buffer/dNTP mix PCR kit, as per manufacturer’s instructions in a 50 μl reaction volume. The PCR thermocycling parameters were: an initial denaturation step of 98^∘^C for 30 s; 25 cycles of: 98^∘^C for 5 s, 65^∘^C for 5 s, and 72^∘^C for 10 s; final extension at 72^∘^C for 5 min; followed by a 4^∘^C hold. PCR products were visualized by gel electrophoresis (1.5% TAE agarose gel) with SYBR Safe (Invitrogen^TM^) DNA gel staining in order to cut out the 16S rRNA gene amplicons. Amplicons were isolated and purified using an E.Z.N.A.^®^ gel extraction kit (Omega Bio-Tek). Subsequently, the samples were pooled together at equal DNA concentrations to make a single Ion Xpress Plus gDNA fragment barcoded library.

**FIGURE 1:**
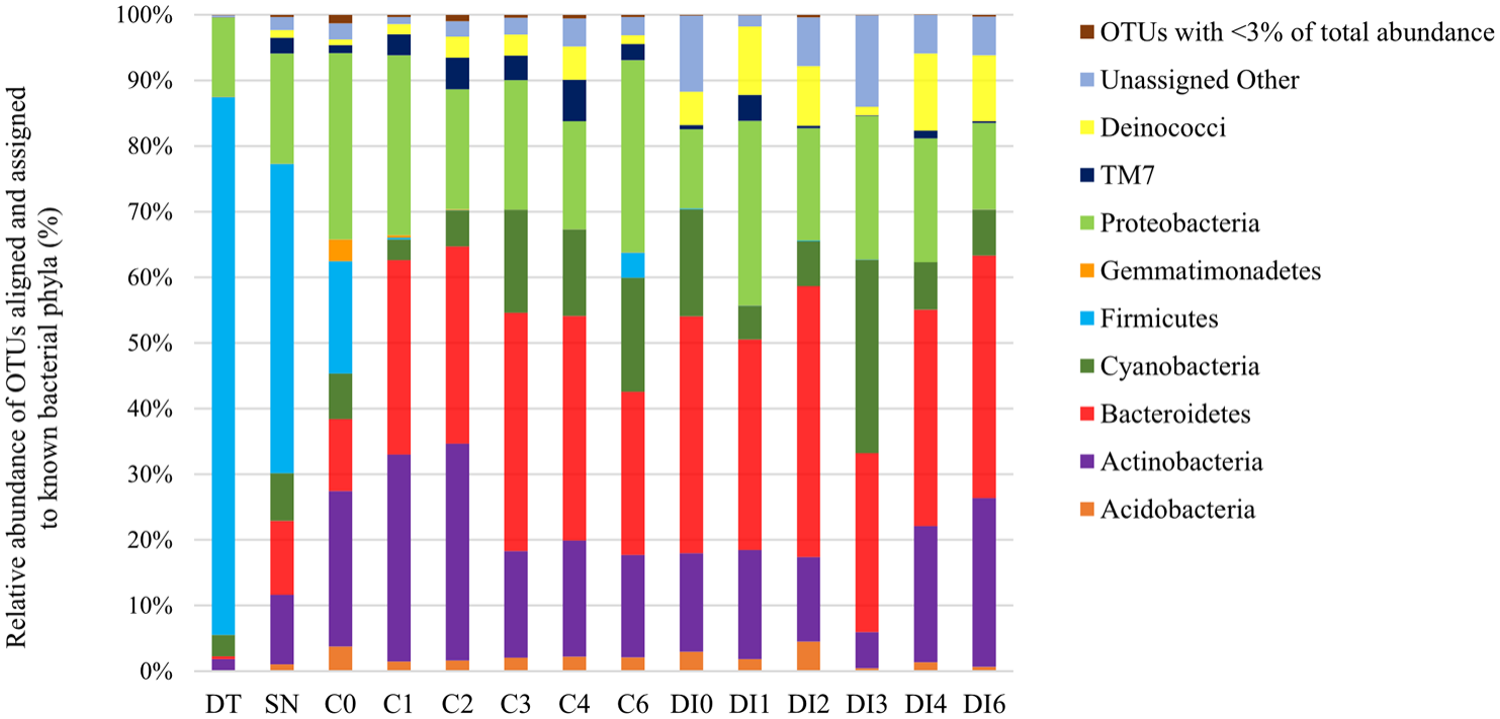
**Percent of bacterial operational taxonomic units (OTUs) aligned and assigned to known bacterial phyla based on PCR amplifications of 16S rRNA gene sequences for all the sample types.** OTUs with <3% of total abundance were grouped together.

Amplicon sequencing of the 16S rRNA genes in the 16S amplicon pool library was prepared using the Ion Plus Fragment Library Kit (Life Technologies). Template preparation and sequencing were carried out on an Ion One Touch 2 and Ion Torrent^TM^ PGM platform Kit (Life technologies), using Ion PGM Template OT2 400 Kit, and Ion PGM sequencing 400 Kit with an Ion 316v2 chip. Data were analyzed using Ion Torrent Suite v4.0.2 software with default filter and quality settings. The 16S rRNA gene sequences were demultiplexed, quality filtered and processed using standard operating procedures in the Quantitative Insights into Microbial Ecology (QIIME) platform (Caporaso et al., 2010b). Bacterial operational taxonomic units (OTUs) were defined as sequences that possessed ≥97% pairwise identity. They were assigned using the UCLUST algorithm against the August 2014 Greengenes reference library (DeSantis et al., 2006; Edgar, 2010) with representative sequences from each OTU selected for taxonomy assignment. The bacterial sequences were aligned using PyNAST (Caporaso et al., 2010a) and classified using the Ribosomal Database Project classifier (RDP; Wang et al., 2007). CHIMERASLAYER (Haas et al., 2011) in QIIME was used to identify chimeric sequences within all sequence profiles and remove them from taxonomical and statistical analyses. Sequence profiles were rarefied to the number of sequences of the smallest sample sequence output within each pool for beta and alpha diversity studies (40385). QIIME’s Chao1, observed species, and phylogenetic diversity (PD) whole tree species richness estimators were used to study alpha diversity. The QIIME platform was used to determine the distance and dissimilarity matrix through UniFrac distances to visualize the ordination and clustering of the bacterial community composition for beta diversity analyses. The ordination patterns based on phylogenetic distance metrics were evaluated using principal coordinate analysis (PCoA). Differences in microbial community composition between the sample types and sampling dates were assessed by non-parametric permutational analysis of variance (PERMANOVA), and similarities (ANOSIM) through importing the sample OTU’s relative abundance matrix (**Figure 1**) into IBM SPSS Statistics 21.

### Microbial Abundance

Total and autofluorescent cell counts were obtained following previously described methods (Telling et al., 2014). As in section “16S rRNA Gene Barcoded Sequencing,” cryoconite hole and DI samples were chosen from six time points spanning the whole ablation season. Three replicates for each sample type and time point were counted separately. Snow from the beginning of the season and dust trap filter samples from the whole season had to be combined, as above, to provide enough material for microbial abundance analyses. Total cell counts were obtained by staining the cells with 4^′^, 6-diamidino-2-phenylindole (DAPI, Sigma), while the photoautotrophic microbes were enumerated using chlorophyll autofluorescence (AF; Telling et al., 2014). Over 300 stained bacterial cells per slide were counted using epifluorescence microscopy and procedural blanks (sterilized Milli-Q water; 1.2 × 10^1^ ± 0.3 × 10^1^ cells mL^-1^ for snow and 9.3 × 10^1^ ± 1.4 × 10^1^ cells g^-1^ for the other samples) were subtracted from the sample counts.

### Stable Carbon Isotopes

Stable isotope analysis was carried out on acidified samples at the NERC Isotope Geosciences Laboratory. It was possible to analyze the dust trap samples from all time points using this method instead of pooling the samples together, as was necessary previously (see 16S rRNA Gene Barcoded Sequencing and Microbial Abundance). GF/F filters were oven dried (60^∘^C) and treated with concentrated HCl fumes under vacuum for 24 h to remove the inorganic carbonates. Filters were redried (60^∘^C), quartered, and packaged in tin cups. Solid samples were oven dried, crushed using a pestle and mortar, and de-carbonated using 5% HCl. Samples were weighed into silver cups and wrapped in tin. Delta ^13^C analyses were performed on a ThermoFinnigan system comprising an Elemental Analyser fitted with a Zero Blank Autosampler and interfaced to a DELTA + XL stable isotope ratio mass spectrometer in continuous flow mode. Calibration of the δ^13^C values was performed by using the VPDB (Vienna Pee Dee Belemnite) standard and reference samples of USGS-40 and IAEA-CH6. Values are reported in the conventional delta (δ) notation (units of ‰) as relative differences between samples and standards. Typical errors for standard materials indicated a precision of 0.1 ‰(1σ) for δ^13^C. Duplicate analysis was carried out on each sample.

## Results

### Comparisons of Bacterial Diversity between Sample Types

In total, 1,586,096 reads were obtained from 14 samples with read sequence lengths of ∼190 (the two negative controls remained negative and did not pass the quality control). The reads from the samples correspond to 9254 different OTUs, of which 9124 passed the quality filtering and QIIME pipeline processing with clustering at the 97% sequence identity level at an abundance of ≥5 reads in the data set (Caporaso et al., 2010b). The number of reads per sample ranged from 40385 (DI6) to 372351 (C6). QIIME’s species richness estimates for alpha diversity study of the samples ranged between: 35–54, 1515–2691, and 753–1365 rarefaction measures for PD whole tree, Chao1, and observed species, respectively. SN had highest species richness for all three rarefaction measurements. The lowest species richness was measured by PD whole tree, Chao1, and observed species in DT (35.92, 1514.60, and 832.40, respectively) and DI (35.11, 1636.45, and 753.27, respectively). PERMANOVA multivariate diversity analyses were applied to test for differences between sample types. Significant differences were found between all four of the sample groups (i.e., dust, snow, ice, and cryoconite holes, pseudo-*F* = 1.75; *p* = 0.001, and the number of permutations = 999). These results were consistent with tests of similarities (ANOSIM), which also showed that the samples types were significantly different (*R* = 0.88, *p* < 0.001 and the number of permutations = 999). On the other hand, there were no significant differences found between the time point samples for cryoconite holes and DI (PERMANOVA: pseudo-*F* = 1.02; *p* = 0.403 and the number of permutations = 999) and the samples within the same habitat type were very similar to each other (ANOSIM: *R* = 0.009, *p* = 0.462 and the number of permutations = 999).

Dust trap and snow samples were dominated by Firmicutes, 82 and 47%, respectively, (the genus *Enterococcus* composed 63 and 62% of the Firmicutes, respectively; **Figure 1**). They also contained a significant presence of Proteobacteria – DT: 12% and SN: 17% (mostly of Comamonadaceae family of Betaproteobacteria) and smaller percentages (<11%) of Actinobacteria, Bacteroidetes, Cyanobacteria, which were also present in the supraglacial samples. Cryoconite hole and DI samples from different time points, spanning the whole season, were dominated by 11–41% Bacteroidetes (in particular Hymenobacter), 12–29% Proteobacteria (Comamonadaceae family of Betaproteobacteria, similar to the dust trap and snow samples), 5–33% Actinobacteria (mostly Salinibacterium part of the Microbacteriaceae family), and 3–29% Cyanobacteria (particularly Chloroplast Stretophyta and the family of Pseudanabaenaceae in Synechococcophycidece). Smaller percentages of Deinococci (<0.1–12%), TM7 (<0.1–6%), Acidobacteria (<0.1–5%), and Gemmatimonadetes (<0.1–3%) were also found present in most of the samples. The C0 cryoconite samples contained a mixture of the diversity present in the snow/dust trap and the remaining samples.

**FIGURE 2:**
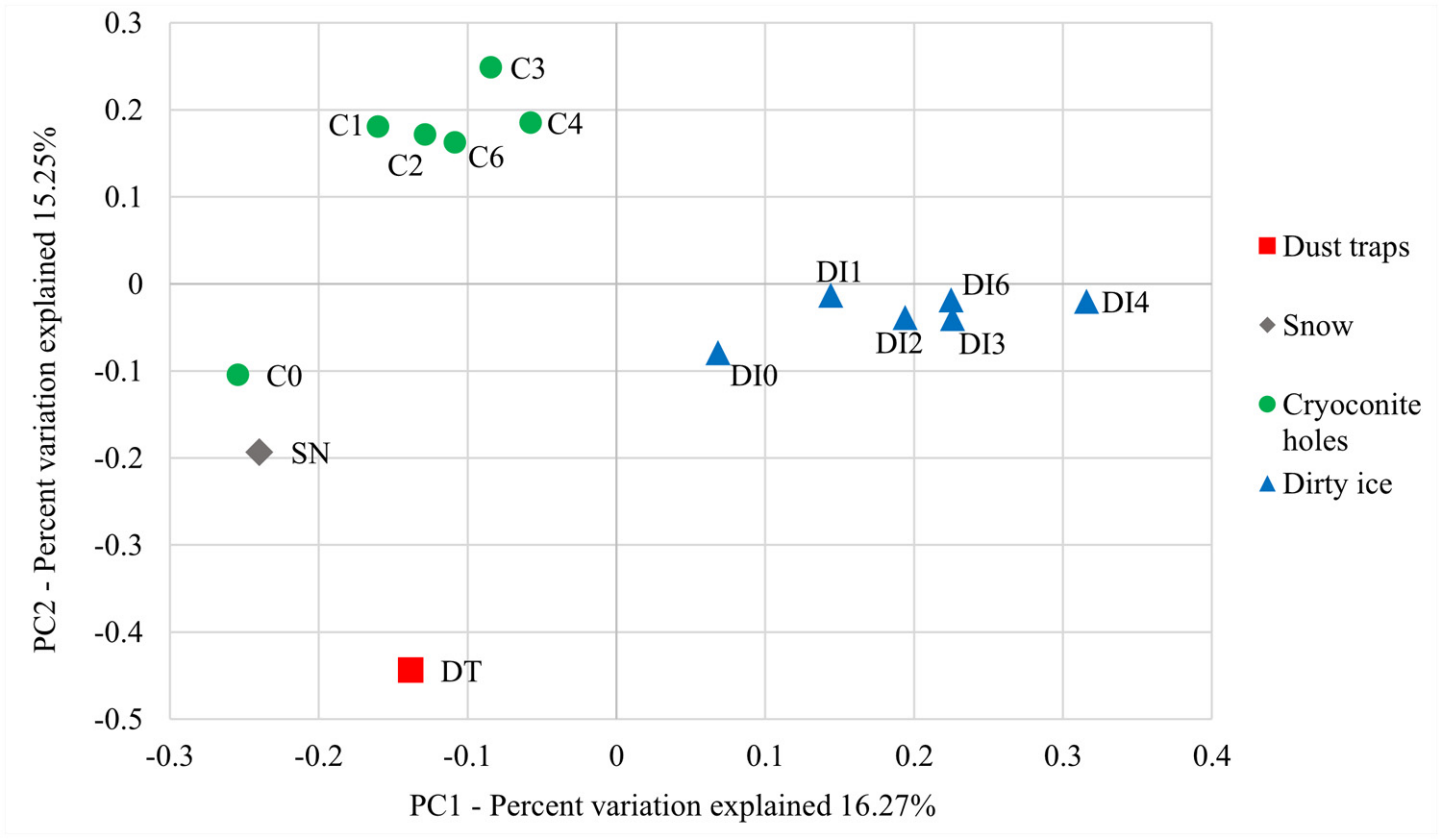
**Principal coordinate analysis (PCoA) ordination patterns based on phylogenetic UniFrac distance metrics.** PC1 is plotted against PC2 to explain 16.27 and 15.24% variation, respectively.

The principal component analyses (PCoA) of ordination patterns reveal four separate sample groupings based on phylogenetic distance metrics (**Figure 2**). C0 samples are more closely related to the snow samples in terms of microbial diversity than the remaining cryoconite time point samples, even though C1 samples were collected less than 2 weeks after the C0 samples. The dust trap diversity related least to the other sample types. Apart from C0, all cryoconite hole samples (C1–C6) are clustered together and equally all DI samples (DI0–DI6) group together. These two groups represent two distinct microbial communities. While there appears to be a progressive change in the microbial communities between samples DI0–DI4 in **Figure 2**, there are no significant differences between sampling time points for neither cryoconite hole nor dirty samples (as above).

### Microbial Abundance

Microbial cell counts of aeolian origin (DT: 6.1 × 10^3^ ± 1.6 × 10^3^ cells g^-1^ and SN: 5.5 × 10^2^ ± 1.2 × 10^2^ cells mL^-1^) were 3–5 orders of magnitude lower than the average of those in cryoconite hole and DI samples, 3.8 × 10^7^ ± 1.0 × 10^7^ cells g^-1^ and 6.0 × 10^6^ ± 1.4 × 10^6^ cells g^-1^, respectively (**Table 1**). The percentage of autotrophic cells in the different samples varied from 5.1 to 17.9% (**Table 1**). Cryoconite hole and DI samples contained the most AF cells (on average 12.4 ± 6.0% and 11.7 ± 4.9%, respectively), while for the dust traps the AF cells only constituted a small percentage (5.1 ± 2.6). There were significant differences between the sample types based on DAPI cell counts (two way ANOVA *p* < 0.001) and AF cell counts (two way ANOVA *p* < 0.001), but not for %AF counts. There were also no significant differences between the DAPI, AF, and %AF counts between sampling time points for cryoconite hole and DI samples. The OTU abundance of Cyanobacteria in all of the sample types (3–29%, **Figure 1**) correlated well (Pearson’s *r* = 0.859, *p* < 0.001) with the %AF cells detected through AF microscopy (5–17%, **Table 1**).

**Table 1:** Microbial cell counts in different samples types and the percent contribution of autofluorescent (AF) cells to the total (DAPI) cells.

Samples	DAPI counts	AF counts	%AF counts
Dust traps	6.1 × 10^3^ ± 1.6 × 10^3^	3.1 × 10^2^ ± 4.2 × 10^1^	5.1 ± 2.6
Snow	5.5 × 10^2^ ± 1.2 × 10^2^	6.1 × 10^1^ ± 1.0 × 10^1^	11.0 ± 8.1
C0	3.8 × 10^7^ ± 9.8 × 10^6^	3.2 × 10^6^ ± 4.2 × 10^5^	8.5 ± 4.3
C1	3.6 × 10^7^ ± 9.6 × 10^6^	2.5 × 10^6^ ± 3.5 × 10^5^	7.0 ± 3.7
C2	3.7 × 10^7^ ± 9.9 × 10^6^	3.8 × 10^6^ ± 3.8 × 10^5^	10.1 ± 3.9
C3	3.9 × 10^7^ ± 1.0 × 10^7^	6.6 × 10^6^ ± 8.8 × 10^5^	17.0 ± 8.5
C4	3.8 × 10^7^ ± 1.0 × 10^7^	5.5 × 10^6^ ± 7.1 × 10^5^	14.3 ± 7.1
C6	4.0 × 10^7^ ± 1.0 × 10^7^	7.1 × 10^6^ ± 8.6 × 10^5^	17.8 ± 8.4
DI0	5.7 × 10^6^ ± 1.2 × 10^6^	9.3 × 10^5^ ± 8.2 × 10^4^	16.3 ± 6.5
DI1	6.2 × 10^6^ ± 1.4 × 10^6^	3.6 × 10^5^ ± 3.4 × 10^4^	5.9 ± 2.4
DI2	6.3 × 10^6^ ± 1.4 × 10^6^	4.1 × 10^5^ ± 3.9 × 10^4^	6.6 ± 2.8
DI3	5.8 × 10^6^ ± 1.2 × 10^6^	1.0 × 10^6^ ± 9.8 × 10^4^	17.9 ± 7.7
DI4	5.9 × 10^6^ ± 1.3 × 10^6^	7.8 × 10^5^ ± 6.9 × 10^4^	13.3 ± 5.2
DI6	6.1 × 10^6^ ± 1.3 × 10^6^	6.1 × 10^5^ ± 6.6 × 10^4^	10.0 ± 4.9


*The counts are in cells mL^-1^ melted snow for the snow samples and cells g^-1^ of debris for the remaining samples. Errors were calculated as 1σ (*n* = 3).*

### Stable Carbon Isotopes

**FIGURE 3:**
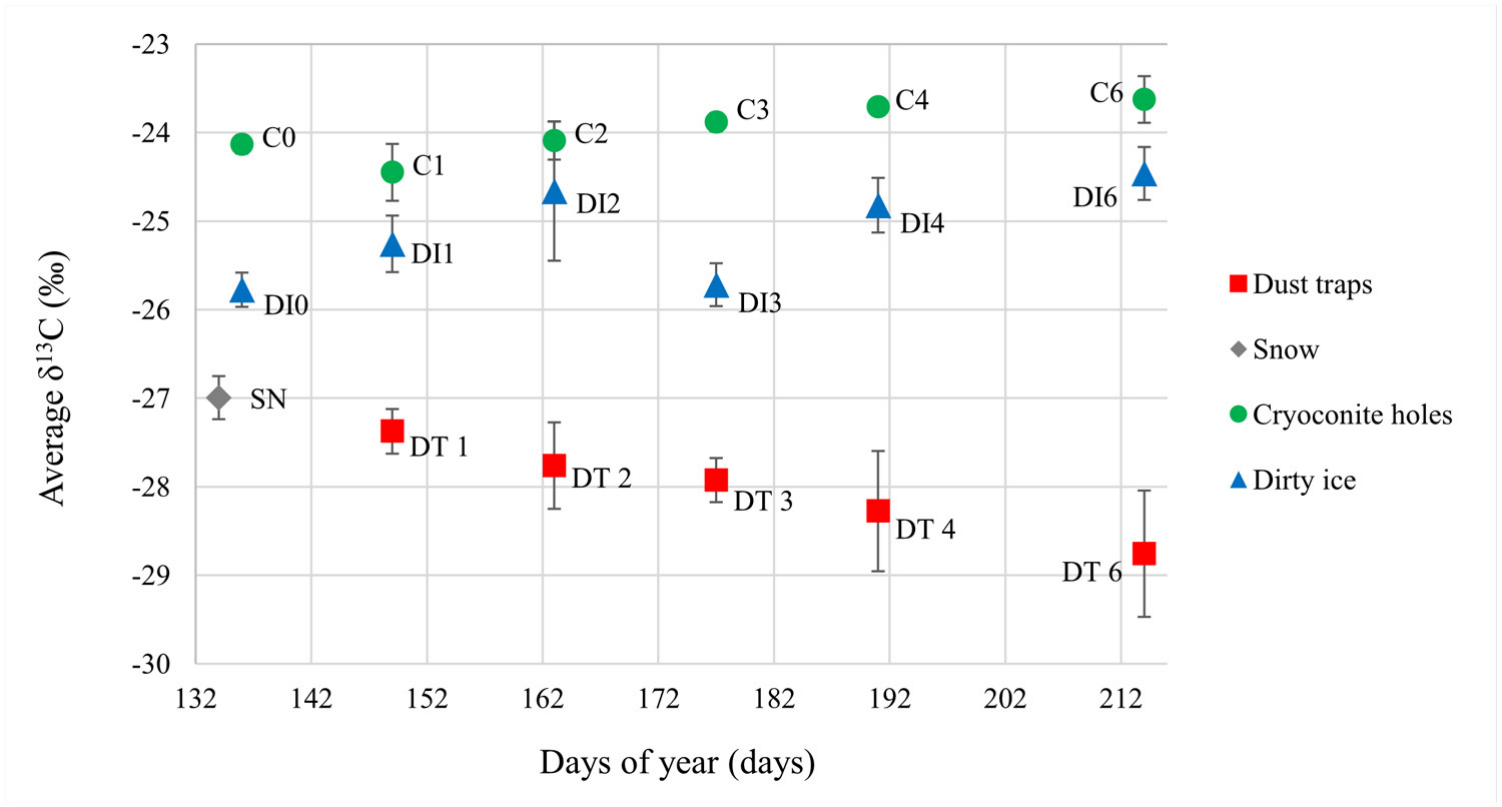
**Average δ^13^C values for all sample types over the 2012 ablation season.** Errors were calculated as 1σ with *n* = 2 for snow and dust trap samples, and *n* = 4 for cryoconite hole (C) and dirty ice (DI) samples.

Dust trap and snow samples had the lowest δ^13^C signature (on average -28.0 ± 0.6‰; δ^13^C and -27.0 ± 0.2‰ δ^13^C, respectively) of the sample set (**Figure 3**). The isotopic values in the cryoconite hole samples increased progressively over the ablation season (from -24.1 ± 0.1to -23.6 ± 0.3‰, Pearson’s *r* = 0.872, *p* < 0.05). DI δ^13^C also appeared to increase throughout the season from -25.8 ± 0.2 to -24.5 ± 0.3‰, apart from an outlier at time point 3. However, there was no significant correlation found between increasing δ^13^C and time, even when the outlier was removed. Significant differences were found between the sample types (two way ANOVA *p* < 0.001).

## Discussion

This study has revealed the inputs of microbes through aeolian transport and snow to microbial communities in cryoconite holes and DI throughout the melt season. We found that supraglacial environments could be seeded by aeolian (wind and snow) microbes, based on their genetic similarities. Aeolian samples contained similar OTUs to those found in all of the other supraglacial samples, such as Proteobacteria, Cyanobacteria, and Actinobacteria (**Figure 1**). Snow samples contained more bacterial phyla than the dust trap samples (e.g., Bacteroidetes, TM7, and Deinococci). In fact, snow samples also had the greatest alpha diversity and therefore the largest pool of microbial communities of all the sample types. Both snow and dust samples were dominated by Firmicutes, which did not establish themselves in the supraglacial communities. It is possible that they were either not adapted to the supraglacial conditions, inactive or dead at the time of inoculation, or they were outcompeted once they were deposited by wind/snow into cryoconite hole and surface ice habitats. Furthermore, microbes of aeolian origin also faced strong competition in terms of cells numbers once they were added to cryoconite holes/DI communities (**Table 1**). On the other hand, bacterial taxa in aeolian dust, such as Proteobacteria, Bacteroidetes, Cyanobacteria, and Actinobacteria, were probably rapidly incorporated into the local microbial communities of these taxa already present on the glacier surface. Alternatively, the genetic similarities between the supraglacial and aeolian samples could be explained by former seeding the latter or that these taxa could be common habitat generalists dominating these environments. Indeed, similar microbial communities have previously been reported in cryoconite and snow samples from Greenland and Svalbard. They include significant amounts of Proteobacteria (Cameron et al., 2012b; Hell et al., 2013; Edwards et al., 2014; Stibal et al., 2015); different types of Bacteroidetes, Actinobacteria, Acidobacteria (Cameron et al., 2012b; Edwards et al., 2014; Stibal et al., 2015), Firmicutes (Amato et al., 2007; Cameron et al., 2012b; Hell et al., 2013; Edwards et al., 2014), and Cyanobacteria (Larose et al., 2010; Cameron et al., 2012b; Edwards et al., 2014; Stibal et al., 2015). The strong correlation between the OTU abundance of Cyanobacteria and percentage of AF cells in all of the sample types is indicative of Cyanobacteria being the major phototrophs in the microbial communities.

The microbial community of cryoconite holes sampled at the very start of the ablation season (C0) potentially appears to be a mixture of aeolian seeding through windborne dust and snow (**Figure 2**), and pre-existing supraglacial communities. Snow samples were collected one day before the snow turned into slush when the temperatures rose and the ablation season started in the study site. Cryoconite holes became uncovered from the snow and the melting snow most likely seeded them with the aeolian microbes. Therefore, the C0 samples collected on the first day of the ablation season would have contained a mixture of previously frozen cryoconite hole microbes and freshly added snow microbes. It is interesting to note that the DI time zero samples (DI0) only contained an insignificant amount of Firmicutes (<0.1%). Aeolian and snow microbes were thus probably washed or blown away from the DI with little opportunity to settle in these habitats. Cryoconite holes, on the other hand, provided a more sheltered environment for these microbes to be retained temporarily within the pre-existing stable community. DI habitats were composed of stable communities that appeared to be distinct from those in cryoconite holes (**Figure 2**). The former contained greater amounts of heterotrophic bacterial phyla (e.g., Deinococcaceae and Bacteroidetes) and all the DI samples clustered into a group separate from the cryoconite hole cluster (**Figure 2**). These differences could be explained by the different supraglacial habitats: (1) cryoconite holes provide protected habitats unlike the more exposed and unstable DI environments; and (2) cryoconite holes may also benefit from greater carry-over effects of stable microbial communities, preserved frozen-in over winter, from the previous melt seasons compared to the DI.

Sampling at regular intervals throughout the whole ablation season also provided unprecedented data on the temporal variability of the structure and abundance of supraglacial microbial communities, as well as the associated organic carbon δ^13^C signature over one summer season (**Figures 2** and 3; **Table 1**). Rapid turnovers were observed between microbial habitats within this study site at the beginning of the summer season. In less than 2 weeks (C1), the dominant microbes in the aeolian samples were reduced to insignificant amounts of OTUs present in the cryoconite holes, even though they appeared to seed the cryoconite holes at the beginning of the season (C0). DI habitats potentially provided even faster turnover times with almost no aeolian/snow microbial communities present at DI0. Once the ablation season started, cryoconite holes and DI remained stable communities for the rest of the season (>65 days). These hypotheses are based on the assumption that there was no (or only minimal) spatial variability between the very similar, neighboring cryoconite holes and DI patches sampled within the small sampling site at each time point (see Material and Methods). Both the *in situ* autotrophic and heterotrophic activity by the DI and cryoconite hole stable communities modified the δ^13^C signature of the aeolian organic carbon added to the supraglacial environments. Samples of aeolian origin had significantly lower δ^13^C values (-28.0 to -27.0‰) from those of the already present supraglacial debris (-25.7 to -23.6‰ **Figure 3**). The low aeolian organic carbon δ^13^C values are probably the result of terrestrial organic carbon transformation at its origin by C3 plants, using the CBB carbon fixation pathway (O’Leary, 1981; Berg et al., 2010), prior to being transported by wind or snow to the glacier surface. An increase in activity over the summer season of the plants and/or organisms at the origin of the aeolian organic carbon is probably responsible for the decreasing δ^13^C values observed in the dust samples. The CBB cycle is also expected to be the dominant carbon fixation pathway for cryoconite autotrophs (Cameron et al., 2012a), yet the organic carbon δ^13^C values were higher. These values are similar to those previously found in Greenlandic and Arctic cryoconite (-23.7 ± 2.2% and -24.6 ± 1.0‰, respectively; Hodson et al., 2010b). The most likely explanation for these higher δ^13^C values is the source of inorganic carbon to the two environments. Higher δ^13^C values are expected in DIC compared to atmospheric CO_2_ (Fogel and Cifuentes, 1993) and cryoconite hole microbes prefer DIC to CO_2_ (Stibal and Tranter, 2007). Cryoconite hole δ^13^C values continued to increase throughout the ablation season despite the continuous additions of dust particles with low δ^13^C values over the summer (**Figure 3**). This is not surprising considering the low input of cells from the dust relative to the numbers of cells in the cryoconite holes (**Table 1**). The progressive increase in ^13^C values of the cryoconite hole samples could result from increasing *in situ* microbial primary productivity and possibly heterotrophic uptake of organic carbon resulting in a +1 increase in δ^13^C values (Deniro and Epstein, 1978), as supported by increasing cell numbers (Pearson’s *r* = 0.770, *p* < 0.001). On the other hand, the more unstable DI habitats, as postulated above, probably led to the observed fluctuations in δ^13^C values. DI environments are more disrupted by washing in and out by ice melt than the sheltered cryoconite holes, which probably affected their *in situ* microbial activity, both autotrophic and heterotrophic, and thus their stable ^13^C signatures. Both cryoconite holes and dirty ice habitats have been found to have continuous microbial activity throughout the 2012 ablation season, at this sampling site, on the GrIS (Musilova et al., in preparation).

## Conclusion

In conclusion, this study elucidated for the first time the aeolian inputs and transformations of GrIS supraglacial microbial communities over the course of a whole ablation season. Aeolian inputs of microbial cells were limited, yet they did potentially seed the local glacier surface microbial habitats with microbes that formed the stable glacier communities. Nevertheless, the dominant bacterial taxa in the aeolian samples did not establish themselves in the cryoconite hole and DI communities. Fast community turnovers were observed between different microbial habitats, particularly at the start of the ablation season after the initial snow melt. However, once the melt season started, the composition of the supraglacial microbial communities remained stable throughout the season. These findings show that future field studies will be able to sample at any time, during one melt season at one study site, without obtaining significant differences in their diversity studies (provided sampling is not conducted within at least 2 weeks of snowmelt). The stable communities also remained active during the summer, making them important contributors to biogeochemical nutrient cycling on glaciers.

## Conflict of Interest Statement

The authors declare that the research was conducted in the absence of any commercial or financial relationships that could be construed as a potential conflict of interest.
